# Characteristics and outcomes of patients admitted to adult intensive care units in Hong Kong: a population retrospective cohort study from 2008 to 2018

**DOI:** 10.1186/s40560-020-00513-9

**Published:** 2021-01-06

**Authors:** Lowell Ling, Chun Ming Ho, Pauline Yeung Ng, King Chung Kenny Chan, Hoi Ping Shum, Cheuk Yan Chan, Alwin Wai Tak Yeung, Wai Tat Wong, Shek Yin Au, Kit Hung Anne Leung, Jacky Ka Hing Chan, Chi Keung Ching, Oi Yan Tam, Hin Hung Tsang, Ting Liong, Kin Ip Law, Manimala Dharmangadan, Dominic So, Fu Loi Chow, Wai Ming Chan, Koon Ngai Lam, Kai Man Chan, Oi Fung Mok, Man Yee To, Sze Yuen Yau, Carmen Chan, Ella Lei, Gavin Matthew Joynt

**Affiliations:** 1 Department of Anaesthesia and Intensive Care, The Chinese University of Hong Kong, 4/F Main Clinical Block and Trauma Centre, Prince of Wales Hospital, Shatin, Hong Kong, China; 2 Department of Anaesthesia and Intensive Care, Tuen Mun Hospital, Hong Kong, China; 3 Department of Medicine, Li Ka Shing Faculty of Medicine, The University of Hong Kong, Hong Kong, China; 4 Department of Adult Intensive Care, Queen Mary Hospital, The University of Hong Kong, Hong Kong, China; 5 Department of Intensive Care, Pamela Youde Nethersole Eastern Hospital, Hong Kong, China; 6 Department of Medicine & Geriatrics, Ruttonjee and Tang Shiu Kin Hospitals, Hong Kong, China; 7 Department of Intensive Care, Queen Elizabeth Hospital, Hong Kong, China; 8 Department of Medicine, Tseung Kwan O Hospital, Hong Kong, China; 9 Department of Intensive Care, Kwong Wah Hospital, Hong Kong, China; 10 Department of Intensive Care, United Christian Hospital, Hong Kong, China; 11 Department of Intensive Care, Princess Margaret Hospital, Hong Kong, China; 12 Department of Intensive Care, Yan Chai Hospital, Hong Kong, China; 13 Department of Intensive Care, Caritas Medical Centre, Hong Kong, China; 14 Department of Intensive Care, North District Hospital, Hong Kong, China; 15 Intensive Care Unit, Department of Medicine, Alice Ho Miu Ling Nethersole Hospital, Hong Kong, China; 16 Quality and Safety Division, Hospital Authority Head Office, Hong Kong, China

**Keywords:** Intensive care, APACHE IV, Benchmarking, Hong Kong, Standard mortality ratio

## Abstract

**Background:**

Globally, mortality rates of patients admitted to the intensive care unit (ICU) have decreased over the last two decades. However, evaluations of the temporal trends in the characteristics and outcomes of ICU patients in Asia are limited. The objective of this study was to describe the characteristics and risk adjusted outcomes of all patients admitted to publicly funded ICUs in Hong Kong over a 11-year period. The secondary objective was to validate the predictive performance of Acute Physiology And Chronic Health Evaluation (APACHE) IV for ICU patients in Hong Kong.

**Methods:**

This was an 11-year population-based retrospective study of all patients admitted to adult general (mixed medical-surgical) intensive care units in Hong Kong public hospitals. ICU patients were identified from a population electronic health record database. Prospectively collected APACHE IV data and clinical outcomes were analysed.

**Results:**

From 1 April 2008 to 31 March 2019, there were a total of 133,858 adult ICU admissions in Hong Kong public hospitals. During this time, annual ICU admissions increased from 11,267 to 14,068, whilst hospital mortality decreased from 19.7 to 14.3%. The APACHE IV standard mortality ratio (SMR) decreased from 0.81 to 0.65 during the same period. Linear regression demonstrated that APACHE IV SMR changed by − 0.15 (95% CI − 0.18 to − 0.11) per year (Pearson’s *R* = − 0.951, *p* < 0.001). Observed median ICU length of stay was shorter than that predicted by APACHE IV (1.98 vs. 4.77, *p* < 0.001). C-statistic for APACHE IV to predict hospital mortality was 0.889 (95% CI 0.887 to 0.891) whilst calibration was limited (Hosmer–Lemeshow test *p* < 0.001).

**Conclusions:**

Despite relatively modest per capita health expenditure, and a small number of ICU beds per population, Hong Kong consistently provides a high-quality and efficient ICU service. Number of adult ICU admissions has increased, whilst adjusted mortality has decreased over the last decade. Although APACHE IV had good discrimination for hospital mortality, it overestimated hospital mortality of critically ill patients in Hong Kong.

**Supplementary Information:**

The online version contains supplementary material available at 10.1186/s40560-020-00513-9.

## Background

Globally, mortality rates of patients admitted to the intensive care unit (ICU) have decreased over the last two decades [1–6]. This is remarkable given the increases in admission age, number of comorbidities and severity of illness of critically ill patients [7]. However, these trends are mostly based on longitudinal data from high-income Western countries. Descriptive data on characteristics and risk adjusted outcomes of critically ill patients in Asia suggests differences in patient severity of illness, length of stay and mortality compared to data from Western countries [8, 9]. This may be partly explained by regional differences in critical care bed capacity associated with differences in national wealth and resources [10].

Evaluations of the temporal trends in the characteristics and outcomes of ICU patients in Asia are limited. Nationwide ICU data from Taiwan and Korea showed increasing numbers of ICU admissions with decreasing mortality between 2009–2014 and 1997–2013, respectively [5, 11]. In contrast, the Malaysian Registry of Intensive Care reported in 2018 a relatively steady number of ICU admissions, with a small and non-significant decrease in adjusted mortality over 5 years [12]. Quantifying and understanding country-specific casemix, patient characteristics and outcomes of critically ill patients is needed to facilitate the design and interpretation of regional clinical trials involving Asian countries and regions. Moreover, appropriate adjustments for country and regional differences in critical care provisions and performance may be needed in large international multicentre trials, which are increasingly common in critical care. Documentation of rigorously adjusted performance data allows comparisons of healthcare systems, such that successful health strategies can be identified and potentially adopted in regions where performance may be lagging [13].

Hong Kong is a special administrative region of China, but has its own independent fiscal budget and healthcare system. As a high-income region with a population of 7.482 million, Hong Kong spent just 6.2% of its gross domestic product (GDP) in health expenditure in 2019 [14]. This equates to a spending of $3061 USD per capita, which is at least three times less than what the USA spends on healthcare. The vast majority (> 90%) of acute care in Hong Kong is provided in publicly funded hospitals, with only a minority of patients receiving care in privately funded institutions [15]. Acute admissions in the public health system are managed across 23 acute hospitals, supported by 15 general adult ICUs (Additional file 1). In addition, the proportion of critical care beds in Hong Kong acute hospitals is less than 3% compared to 12% in the USA [16, 17]. Public ICUs in Hong Kong are uniformly staffed by 4.2 nurses and 0.9 doctors employed per functional bed. Across Hong Kong, 60% of doctors and 65% of nurses staffing the ICUs have specialist qualifications in intensive care.

Utilising internationally validated scoring systems such as the Acute Physiology and Chronic Health Evaluation (APACHE) score, single-centre studies from Hong Kong suggest that ICU patients in Hong Kong have better than expected mortality and length of stay, but have not improved substantially over time [18–20]. The objective of this study was to describe the characteristics and risk adjusted outcomes of all patients admitted to all publicly funded ICUs in Hong Kong over a 11-year period. The secondary objective was to validate the predictive performance of APACHE IV for ICU patients in Hong Kong.

## Methods

### Study design and cohort selection

This was an 11-year population-based retrospective study of all patients admitted to adult general (mixed medical-surgical) intensive care units in Hong Kong public hospitals. All adult (≥ 18 years old) ICU patients admitted to Hong Kong public hospitals between 1 April 2008 and 31 March 2019 were included. Only the first ICU admission for each patient hospital episode was included, defined as admission to hospital until discharge home or death. If patients were transferred between ICUs, only data from the first ICU admission was included. This study was approved by The Joint Chinese University of Hong Kong–New Territories East Cluster Clinical Research Ethics Committee with waiver of informed consent (2020.078) and the local ethics committees of each participating ICU.

### Data collection

Since 2008, all units have prospectively collected the complete APACHE IV dataset on all admitted patients for the purpose of clinical audit and benchmarking. ICU mortality, hospital mortality, ICU length of stay (LOS), hospital LOS and discharge destination were recorded. All APACHE IV data are collected by trained nurses and validation of data collection is performed annually by a central audit team. Data is stored in a population electronic health database called the Clinical Data Analysis and Reporting System (CDARS) of the Hospital Authority. CDARS also contains admission and discharge data, diagnosis and procedure codes, medication records, operation records, laboratory and microbiology results of all outpatient and inpatients treated in Hong Kong public hospitals since 1995. Hong Kong population census data, information on public hospital admissions, bed capacity and mortality were collected from the Census and Statistics Department of the Government of the Hong Kong and Hospital Authority’s Annual Statistical Reports. Adult public hospital acute beds were calculated by excluding paediatric, rehabilitation and palliative care beds.

### Data analysis

Descriptive statistics such as frequencies and percentages were used for categorical variables. Normality for continuous variables was assessed with Shapiro-Wilk test and expressed as means and standard deviations or medians and interquartile range as appropriate.

Annual rates of ICU admission per population were expressed per 10,000 population. It was calculated from the number of ICU admissions divided by the mid-year usual resident population of Hong Kong for that year. Annual rates of ICU admission per hospital were expressed per 10,000 hospital admissions and calculated from the number of ICU admissions divided by the total number of public hospital admissions for that year. APACHE IV mortality discrimination was assessed using C-statistics. To assess APACHE IV calibration in our cohort, Hosmer–Lemeshow test and calibration plot of expected against observed mortality was used [21, 22]. Calibration plot was constructed by dividing the entire cohort into equal deciles of APACHE IV predicted mortality and plotting the observed mortality within each risk group. The difference between the observed and the APACHE IV predicted median ICU LOS was compared using Wilcoxon signed-rank test. The standardised mortality rate (SMR) was calculated by the number of actual hospital deaths divided by APACHE IV predicted deaths for each calendar year. The SMR and C-statistics trend during the study period was assessed using linear regression and Pearson’s correlation.

## Results

### Population and admission trends

The mid-year usual resident population of Hong Kong increased from 6,957,800 to 7,451,000 between 2008 and 2019 (Table 1). The proportion of those age ≥ 60 years old increased by 42.7% during the same period [23]. There were consistent annual increases in ICU and hospital admissions, although there was a reduction in crude hospital mortality over the same period. In 2008, there were 14,442 acute adult hospital beds, of which 240 (1.66%) were ICU/high dependency unit (HDU) beds. By 2019, 249/16,418 (1.52%) of acute adult hospital beds were made up of general ICU/HDU beds (Additional file 1).

**Table 1:** Hong Kong population and annual public hospital and ICU admissions

	2008	2009	2010	2011	2012	2013	2014	2015	2016	2017	2018
**Hong Kong population**	6,957,800	6,972,800	6,832,500	6,886,800	6,949,700	6,986,200	7,022,600	7,079,800	7,146,400	7,391,700	7,451,000
**Proportion of population age ≥ 80 years old**	3.3	3.5	3.7	3.9	4.0	4.2	4.4	4.5	4.7	4.8	4.9
**ICU admissions**	11,267	11,341	11,141	11,696	12,122	11,578	11,022	12,781	13,195	13,647	14,068
**ICU admission per 10,000 population**	16	16	16	17	17	17	16	18	18	18	19
**Hospital admissions**	867,805	907,270	936,743	958,981	983,756	989,709	1,016,499	1,037,616	1,086,443	1,123,019	1,118,657
**ICU admissions per 10,000 hospital admissions**	130	125	119	122	123	117	108	123	121	121	126
**Crude hospital mortality (per 1000 patients)**	25.4	24.5	23.8	22.8	22.4	23.2	22.4	22.6	20.9	20.7	20.7
**ICU occupancy (%)**	74.8	73.8	73.6	73.5	75.8	77.3	78.5	78.7	80.1	79.6	80.6

Population structure of Hong Kong during 2008 to 2018 and annual admission statistics to public hospitals and ICUs

*ICU* intensive care unit

### Cohort demographics and outcomes

**Fig. 1:**
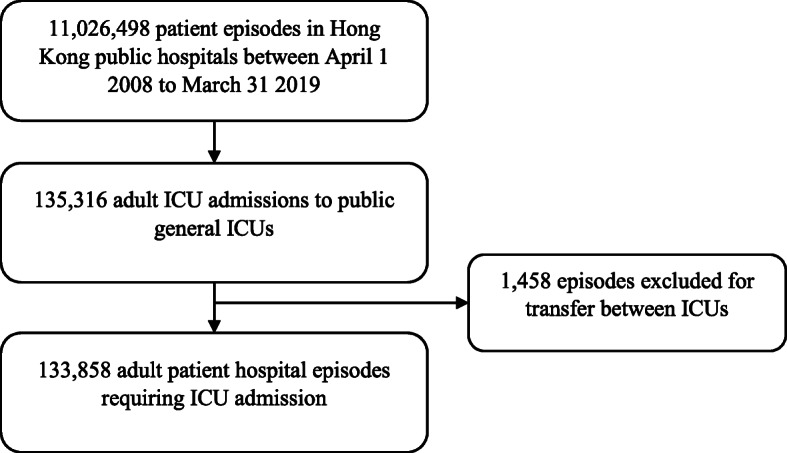
Cohort identification

**Fig. 2:**
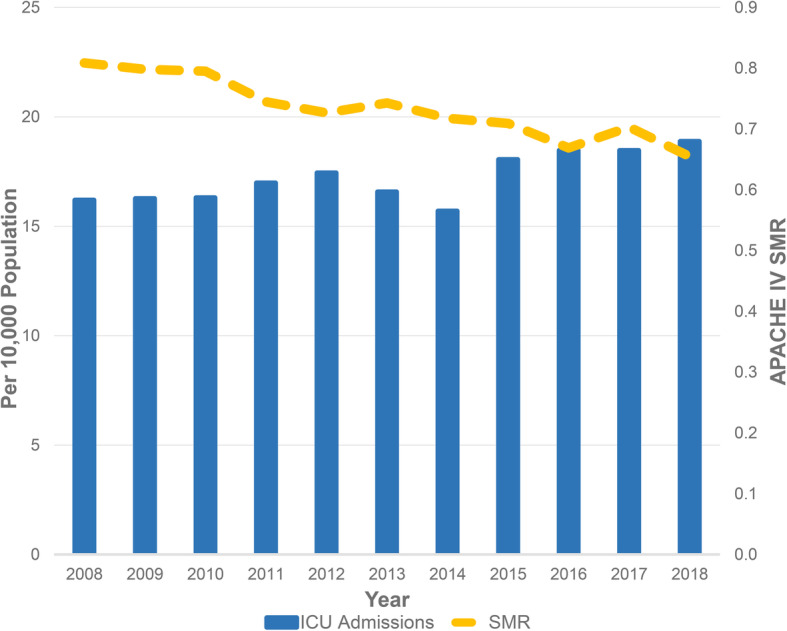
Trend in ICU admissions with APACHE IV SMR. This graph shows increasing ICU admissions per 10,000 population but decreasing APACHE IV SMR from 2008 to 2018. APACHE, Acute Physiology and Chronic Health Evaluation; SMR, standardised mortality ratio

From 1 April 2008 to 31 March 2019, there were a total of 133,858 adult patient episodes that required admission to ICU (Fig. 1). The trends in ICU patient demographics and outcomes are shown in Table 2. Hospital discharge destination of hospital survivors is shown in Additional file 2. Information on discharge destination was missing in 54 patients. The severity of illness and outcomes differed between medical, elective post-operative and emergency post-operative patients (Additional file 3). The trends in SMR from 2008 to 2018 along with ICU and hospital admission rates are shown in Fig. 2. Linear regression demonstrated that APACHE IV SMR changed by − 0.15 (95% CI − 0.18 to − 0.11) per year (Pearson’s *R* = − 0.951, *p* < 0.001). Trends in admission diagnosis categories are given in Additional file 4.

**Table 2:** ICU patient characteristics and outcomes

	2008	2009	2010	2011	2012	2013	2014	2015	2016	2017	2018
**Age (years)**	63 (50–75)	62 (50–75)	63 (50–75)	62 (50–75)	62 (50–75)	62 (51–75)	63 (51–74)	63 (52–75)	63 (52–74)	64 (52–74)	63 (52–73)
**Age ≥ 80 years (%)**	14.3	13.3	14.5	14.7	14.2	14.5	14.1	15.3	15	15	13.2
**Male gender (%)**	61.8	61.8	61.2	61.4	60.5	61.4	62.0	61.9	61.7	61.9	60.4
**Comorbidity (%)**	13.3	14.5	13.5	12.4	15.9	13.7	15.1	15.7	15.6	16.1	17.4
**Admission type**											
**Elective surgery (%)**	23.0	21.4	20.3	21.9	24.3	24.5	24.8	23.1	22.7	21.6	22.0
**Emergency surgery (%)**	21.2	21.3	22.9	24.4	21.8	21.5	22.3	21.7	20.5	20.9	20.9
**Medical (%)**	55.8	57.3	56.9	53.7	53.9	54.0	52.9	55.2	56.8	57.5	57.1
**APACHE IV APS**	51 (33–76)	51 (33–76)	50 (34–74)	49 (32–73)	49 (32–73)	49 (32–75)	49 (32–75)	49 (32–74)	49 (32–74)	49 (32–72)	48 (32–71)
**APACHE IV**	64 (43–89)	63 (43–89)	63 (43–87)	61 (42–86)	62 (42–87)	61 (42–88)	61 (42–87)	62 (42–88)	62 (43–88)	61 (43–85)	61 (43–84)
**Mechanical ventilation (%)**	55.1	56.2	56.5	55.1	53.9	54.8	53.4	54.0	51.8	51.6	53.3
**Vasopressor (%)**	12.1	12.1	11.7	12.3	13.1	14.7	16.4	24.0	23.4	27.1	30.9
**Renal replacement therapy (%)**	9.6	11.6	13.2	12.1	11.5	11.8	12.8	13.1	13.1	14.1	13.2
**ICU LOS (days)**	2.0 (1–4.4)	1.9 (1.0–4.1)	2.0 (1.0–4.5)	1.9 (1.0–4.2)	2.0 (1.0–4.5)	2.0 (1.0–4.6)	2.0 (1.0–4.6)	2.0 (1.0–4.7)	2.0 (1.0–4.5)	2.0 (1.0–4.4)	2.0 (1.0–4.2)
**Predicted ICU LOS (days)**	4.9 (3.1–6.6)	4.9 (3.2–6.6)	4.9 (3.2–6.7)	4.8 (3.2–6.6)	4.7 (3.1–6.6)	4.7 (3.1–6.6)	4.7 (3.0–6.6)	4.7 (3.1–6.7)	4.7 (3.0–6.7)	4.7 (3.0–6.7)	4.7 (3.0–6.6)
**Hospital LOS (days)**	12.2 (6.8–24.4)	12.1 (6.5–24.1)	11.9 (6.5–24.0)	12.1 (6.7–24.1)	12.2 (6.8–24.2)	12.4 (6.8–24.1)	13.0 (7.0–25.6)	12.5 (6.8–25.8)	12.8 (6.9–25.8)	13.2 (6.9–27.0)	13.0 (7.1–26.2)
**ICU mortality (%)**	12.5	12.1	12.0	11.0	10.8	10.7	9.8	9.7	9.4	9.3	8.5
**Hospital mortality (%)**	19.7	19.5	18.8	16.9	17.0	17.6	16.7	16.7	15.7	15.8	14.3
**APACHE IV predicted mortality (%)**	24.3	24.5	23.6	22.7	23.4	23.6	23.2	23.6	23.5	22.5	21.9
**SMR**	0.81	0.80	0.80	0.74	0.73	0.74	0.72	0.71	0.67	0.70	0.65
**Readmission rate (%)**	5.69	5.76	5.48	5.14	4.65	4.87	4.92	5.54	4.96	5.45	5.69

All values in median (IQR) unless otherwise specified. Comorbidity is calculated by having any of the chronic illnesses diagnosis in APACHE IV score. *APACHE* Acute Physiology and Chronic Health Evaluation, *APS* Acute Physiology Score, *LOS* length of stay, *SMR* standardised mortality ratio

### APACHE IV predictive performance

**Fig. 3:**
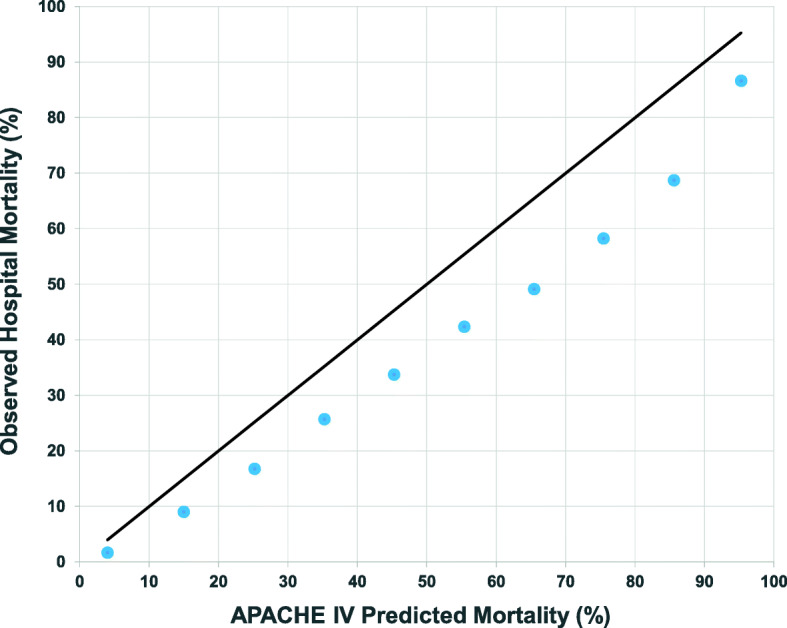
Calibration of APACHE IV on hospital mortality prediction. The reference bold line shows perfect calibration between APACHE IV predicted hospital mortality and hospital mortality. The blue points represent observed hospital mortality. Observed mortality was lower than predicted mortality across the spectrum of illness severity in Hong Kong publicly funded intensive care units

The C-statistic for APACHE IV to predict hospital mortality for the entire cohort was 0.889 (95% CI 0.887 to 0.891) (Additional file 5). APACHE IV C-statistic ranged from 0.883 to 0.897 during the study period. Linear regression of APACHE IV C-statistic showed lack of correlation with time (Pearson’s *R* = 0.198, *p* = 0.559). The Hosmer–Lemeshow test (*p* < 0.001) suggests calibration of the APACHE IV hospital mortality prediction in our cohort was limited. In addition, observed mortality was consistently lower than APACHE IV predicted mortality across the range of severity of illness (Fig. 3 and Additional file 6). Overall observed median ICU LOS was shorter than that predicted by APACHE IV (1.98 vs. 4.77 days, *p* < 0.001). The median ICU adjusted LOS ratio for the entire cohort was 0.52 (IQR 0.30 to 0.96).

## Discussion

In this 11-year population-based study of all public hospital ICUs in the territory of Hong Kong, we found an expanding and increasingly elderly population, and that the number of public hospital ICU admissions has correspondingly increased by 25%. Despite the increasing elderly population, the median age and severity of illness of critically ill patients admitted to ICU has remained relatively unchanged, while the hospital mortality of ICU patients has decreased by 32% between April 1 2008 and March 31 2019. When compared with international data, severity of illness adjusted mortality as assessed by APACHE IV SMR is relatively low, as is the predicted LOS ratio, suggesting that admitted cases receive efficient and high-quality care [8, 24–29]. Although the APACHE IV score had good discrimination, it overestimated the hospital mortality for ICU patients in Hong Kong.

The steady increase in ICU admissions in Hong Kong over the last decade is consistent with rising global demands for critical care provision and in proportion to the increased number of hospital admissions [1–6]. Age at admission and the severity of illness of ICU patients have remained relatively constant, with a small increase in the number of admissions suffering from at least one comorbidity. Despite a 48% increase in the proportion of those 80 years or older in the Hong Kong population, this did not translate to a higher proportion of ICU patients who were ≥ 80 years old [23]. In contrast, most longitudinal studies have found an increase in ICU admission age over time, particularly those aged ≥ 80 years old [2–7, 30–32]. Moreover, our median APACHE IV scores of 61 to 64 were higher than severity of illness reported elsewhere [4, 8, 24, 33, 34]. More than 50% of ICU patients in Hong Kong required mechanical ventilation, which is comparable to large cohorts from Spain and UK but much higher than the 28% reported from Korea and USA [2, 4, 11, 35]. Similarly, use of renal replacement therapy in Hong Kong ICU patients was about 5 times more common than those admitted to ICUs in the USA. Interestingly, the use of vasopressors increased threefold over the last decade in Hong Kong ICUs, and is consistent with vasopressor usage trends from the USA [36]. The cause of this trend warrants further investigation but may be due to changes in both case-mix and clinical practice [37].

These particular differences in the evolution of the characteristics of our ICU cohort are possibly accounted for by the relatively limited critical care resources available and the resultant universal practice of ICU admission triage. Hong Kong has one of the lowest critical care beds per population amongst high-income countries internationally and in Asia [10, 38]. Compared to Taiwan, the USA and Germany, Hong Kong has 4 times less available critical care beds. Given this limited ICU bed capacity, prioritising the allocation of critical care beds by triage is required. A single-centre study in Hong Kong found that ICU admission refusals due to triage decisions accounted for 34% of all ICU referrals [39]. A separate study from another Hong Kong ICU noted that 38% of critical care referrals were refused for admission (17% because of triage, 13% because of perceived futility and 8% because patients considered too well for ICU) [40]. Triage decisions in different countries are based on cultural, healthcare structure and resources, and political factors [41]. The constant age and APACHE IV severity of our ICU cohort are likely the result of systematic triage, which is required for increasing absolute number and proportion to population of ICU patients. The increasingly high occupancy rates may influence admission decisions where relatively younger patients with less comorbidities and judged to have the greatest chance of recovery are admitted to critical care.

Limited ICU bed capacity and the consistently high ICU occupancy rates across Hong Kong may partially explain the need for efficiency and the corresponding short ICU LOS. This finding is in keeping with observations from other studies where countries with less ICU bed capacity tend to have shorter ICU LOS [2, 5, 24, 38]. It is worth noting that this efficiency is confirmed by a low APACHE IV adjusted LOS ratio. Furthermore, it did not come at a cost of premature discharge or quality of care, as our re-admission rates are acceptable by international standards, and the SMR comparatively good [42].

Similar to the findings of other international longitudinal studies, mortality rates of Hong Kong ICU patients have also improved over time [1–6]. Furthermore, the proportion of hospital survivors discharged home remained constant without relative increase in discharge to rehabilitation, hospice or nursing facilities. This is important to assess since transfer to nursing facilities and hospices has been shown to bias hospital mortality estimation [35, 43, 44]. Although the improvement in ICU and hospital mortality may simply reflect more effective ICU triage, and hence admission bias, systematic improvement in healthcare delivery is more likely, since crude hospital mortality has also improved. Lastly, the APACHE IV data presented was prospectively collected for local ICU benchmarking. Although not assessed in this study, identification of local outlier ICUs may have contributed to improvements in mortality rates over time [45].

International reports of APACHE IV SMR in mixed ICUs range from 0.67 to 2.85 [8, 24–29]. Hong Kong’s overall APACHE IV SMR of 0.65 to 0.81 over the last decade was consistently at the lower end of reported figures. The reduction in SMR over time was also encouraging, but is a recognised feature of predictive model performance fade over time [46, 47]. Nevertheless, the reduction in mortality over time occurred despite relatively constant APACHE IV mortality discriminative performance. Combining the APACHE IV SMR, ICU LOS ratio, re-admission and home discharge rate data suggests that relative to internationally published data, ICU care in Hong Kong is both high-quality and efficient. These findings also exist in the context of low per capita GDP spending, low ICU bed to population and low ICU bed to hospital bed ratios [48].

Similar to other APACHE IV external validation studies, we found that when the score was applied to our Hong Kong ICU patients it had good discrimination but calibration was less robust [25, 29, 49]. Since our study cohort was large, a significant Hosmer–Lemeshow test may not invariably mean calibration of APACHE IV on Hong Kong ICU patients is weak [21, 22]. However, comparison of expected against observed mortality showed that APACHE IV overestimated mortality of Hong Kong ICU patients across the full spectrum of severity of illness (Fig. 3 and Additional file 6). This may be because of case mix differences, as well as intrinsic deficiencies. Triage may introduce systematic case selection bias if patients are only admitted to ICU if they have good pre-admission functional status. This may result in relatively low SMR in Hong Kong ICU patients across the full range of APACHE IV score as it does not capture frailty. Subsequent mortality predictive models for ICU patients may consider to capture frailty assessment which has been shown to impact APACHE IV mortality prediction [50]. While universal adoption of a single predictive model such as APACHE IV facilitates international benchmarking, local models, such as the Intensive Care National Audit & Research Centre (ICNARC), Australian and New Zealand Risk of Death (ANZROD) and in Hong Kong, Intensive Care Unit Outcomes Monitoring and Improvement Program (ICUOMP), are better calibrated for local use and therefore may be more useful to evaluate detailed national practice and make local comparisons [45, 51].

This study has limitations. First, we were unable to capture admission and outcomes of patients admitted to private ICUs. However, as > 90% of ICU care is provided by public hospitals, our study on all public general adult ICUs should provide a sufficiently robust picture of the ICU population characteristics in the territory. This contrasts to the USA ICUs where scoring system data are captured in 10–15% of all ICU patients [52]. Second, we were unable to include data from one dedicated neurosurgical and one cardiothoracic surgical ICU since APACHE IV data were not routinely collected. Third, this was a retrospective analysis and therefore susceptible to bias. However, bias was likely minimised because we used prospectively collected benchmarking data, which was independently checked and verified, and included the entire population of critically ill patients admitted to all public hospital adult general ICUs.

## Conclusion

Compared with reported international benchmarks, despite relatively modest per capita health expenditure, and a small number of ICU beds per population, Hong Kong consistently provides a high-quality and efficient ICU service. During the last decade, the number of adult ICU admissions has increased, whilst mortality has decreased. Admission age and severity of illness has remained relatively unchanged. Although APACHE IV had good discrimination for hospital mortality, it overestimated hospital mortality of critically ill patients in Hong Kong.

## Supplementary Information

**Additional file 1: Supplementary Table 1.** Adult General ICUs in Hong Kong Public Hospitals in 2019. Acute adult hospital beds were calculated from total hospital beds excluding paediatric, rehabilitation and palliative care beds.

**Additional file 2: Supplementary Table 2.** Discharge destination in hospital survivors. *Includes patients who were discharged against medical advice. Discharge destination was missing in 54 patients.

**Additional file 3: Supplementary Table 3.** Outcomes by reason for ICU admission. Values are in median and (interquartile range) unless specified. APACHE, Acute Physiology and Chronic Health Evaluation; APS, Acute Physiology Score; LOS, length of stay; SMR, standardized mortality ratio.

**Additional file 4: Supplementary Table 4.** Trends in Admission Diagnosis Category. Description: Admission diagnosis were categorized into different systems. *Sepsis was coded when patient had proven or suspected infection with ≥2 systemic inflammatory response syndrome criteria.

**Additional file 5: Supplementary Figure 1.** Receiver operating characteristic curve of APACHE IV on Hospital Mortality. Discrimination performance of APACHE IV score on hospital mortality of critically ill patients in Hong Kong.

**Additional file 6: Supplementary Table 5.** Comparison of APACHE IV Predicted and Observed Hospital Mortality. The study cohort was divided into 10 deciles of APACHE IV predicted mortality groups. Observed number of hospital deaths was compared with APACHE IV predicted number of deaths in each group. 510 patients did not have APACHE IV predicted mortality due to missing data and were not included in this table. APACHE, Acute Physiology and Chronic Health Evaluation.

## Data Availability

The datasets used and/or analysed in this study are available from the corresponding author on reasonable request. Approval from all cluster ethics committees that approved this study will be required before data can be shared.
