# Supplementary material for: Characteristics and outcomes of patients admitted to adult intensive care units in Hong Kong: a population retrospective cohort study from 2008 to 2018
Source: J Intensive Care. 2021 Jan 6;9:2. doi: 10.1186/s40560-020-00513-9 (PMC7788755; doi:10.1186/s40560-020-00513-9)
Supplement: Supplementary file 1 — Additional file 1: Supplementary Table 1. Adult General ICUs in Hong Kong Public Hospitals in 2019. Acute adult hospital beds were calculated from total hospital beds excluding paediatric, rehabilitation and palliative care beds. [file 40560_2020_513_MOESM1_ESM.docx]

**Supplementary Table 1 Adult General ICUs in Hong Kong Public Hospitals in 2019**

| **Hospital** | **Type** | **Hospital Beds** | **ICU/HDU Beds** |
| --- | --- | --- | --- |
| Pamela Youde Nethersole Eastern Hospital | Acute general hospital | 1708 | 23 |
| Ruttonjee & Tang Shiu Kin Hospitals | Acute general hospital | 449 | 8 |
| Queen Mary Hospital | University teaching hospital | 1438 | 20 |
| Queen Elizabeth Hospital | Acute general hospital | 1690 | 24 |
| Kwong Wah Hospital | Acute general hospital | 966 | 13 |
| United Christian Hospital | Acute general hospital | 1316 | 22 |
| Tseung Kwan O Hospital | Acute general hospital | 710 | 14 |
| Caritas Medical Centre | Acute general hospital | 926 | 16 |
| Princess Margaret Hospital | Acute general hospital | 1423 | 20 |
| Yan Chai Hospital | Acute general hospital | 718 | 8 |
| Tuen Mun Hospital | Acute general hospital | 1756 | 26 |
| Pok Oi Hospital | Acute general hospital | 660 | 8 |
| Alice Ho Miu Ling Nethersole Hospital | Acute general hospital | 501 | 9 |
| North District Hospital | Acute general hospital | 646 | 15 |
| Prince of Wales Hospital | University teaching hospital | 1511 | 23 |

Acute adult hospital beds were calculated from total hospital beds excluding paediatric, rehabilitation and palliative care beds.
