# Supplementary material for: Characteristics and outcomes of patients admitted to adult intensive care units in Hong Kong: a population retrospective cohort study from 2008 to 2018
Source: J Intensive Care. 2021 Jan 6;9:2. doi: 10.1186/s40560-020-00513-9 (PMC7788755; doi:10.1186/s40560-020-00513-9)
Supplement: Supplementary file 2 — Additional file 2: Supplementary Table 2. Discharge destination in hospital survivors. *Includes patients who were discharged against medical advice. Discharge destination was missing in 54 patients. [file 40560_2020_513_MOESM2_ESM.docx]

**Supplementary Table 2 Discharge destination in hospital survivors**

|  | **2008** | **2009** | **2010** | **2011** | **2012** | **2013** | **2014** | **2015** | **2016** | **2017** | **2018** |
| --- | --- | --- | --- | --- | --- | --- | --- | --- | --- | --- | --- |
| **Home* (%)** | 77.2 | 75.6 | 77.1 | 78.1 | 77.7 | 79.1 | 78.8 | 77.8 | 78.0 | 78.1 | 77.3 |
| **Rehabilitation Hospital (%)** | 18.9 | 20.1 | 18.1 | 17.4 | 16.6 | 15.3 | 15.5 | 16.3 | 16.2 | 16.1 | 15.8 |
| **Other Acute Hospital (%)** | 3.6 | 4.1 | 4.4 | 4.4 | 5.4 | 5.4 | 5.5 | 5.8 | 5.7 | 5.6 | 6.6 |

*Includes patients who were discharged against medical advice. Discharge destination was missing in 54 patients.
