# Supplementary material for: Characteristics and outcomes of patients admitted to adult intensive care units in Hong Kong: a population retrospective cohort study from 2008 to 2018
Source: J Intensive Care. 2021 Jan 6;9:2. doi: 10.1186/s40560-020-00513-9 (PMC7788755; doi:10.1186/s40560-020-00513-9)
Supplement: Supplementary file 3 — Additional file 3: Supplementary Table 3. Outcomes by reason for ICU admission. Values are in median and (interquartile range) unless specified. APACHE, Acute Physiology and Chronic Health Evaluation; APS, Acute Physiology Score; LOS, length of stay; SMR, standardized mortality ratio. [file 40560_2020_513_MOESM3_ESM.docx]

**Supplementary Table 3 Outcomes by reason for ICU admission**

|  | **Elective Post-op** | **Emergency Post-op** | **Medical** |
| --- | --- | --- | --- |
| **Age (years)** | 63 (54-72) | 64 (51-77) | 62 (50-75) |
| **APACHE IV** | 45 (34-58) | 61 (44-82) | 73 (50-100) |
| **ICU LOS (days)** | 1 (0.8-1.9) | 2.4 (1.3-5.0) | 2.6 (1.3-5.5) |
| **ICU Mortality (%)** | 0.8 (95%CI 0.7-0.9) | 8.6 (95%CI 8.2-8.9) | 15.1 (95%CI 14.9-15.3) |
| **Hospital Mortality (%)** | 3.1 (95%CI 2.9-3.3) | 16.8 (95%CI 12.4-17.3) | 22.8 (95%CI 22.5-23.1) |
| **SMR** | 0.46 (95%CI 0.45 to 0.46) | 0.80 (95%CI 0.79 to 0.80) | 0.74 (95%CI 0.73 to 0.74) |

Values are in median and (interquartile range) unless specified. APACHE, Acute Physiology and Chronic Health Evaluation; APS, Acute Physiology Score; LOS, length of stay; SMR, standardized mortality ratio.
