# Supplementary material for: Characteristics and outcomes of patients admitted to adult intensive care units in Hong Kong: a population retrospective cohort study from 2008 to 2018
Source: J Intensive Care. 2021 Jan 6;9:2. doi: 10.1186/s40560-020-00513-9 (PMC7788755; doi:10.1186/s40560-020-00513-9)
Supplement: Supplementary file 4 — Additional file 4: Supplementary Table 4. Trends in Admission Diagnosis Category. Description: Admission diagnosis were categorized into different systems. *Sepsis was coded when patient had proven or suspected infection with ≥2 systemic inflammatory response syndrome criteria. [file 40560_2020_513_MOESM4_ESM.docx]

|  | **2008** | **2009** | **2010** | **2011** | **2012** | **2013** | **2014** | **2015** | **2016** | **2017** | **2018** |
| --- | --- | --- | --- | --- | --- | --- | --- | --- | --- | --- | --- |
| **Cardiovascular (%)** | 16.2 | 16.2 | 16.2 | 16.1 | 15.9 | 17.0 | 16.0 | 15.8 | 14.9 | 14.7 | 15.5 |
| **Gastrointestinal (%)** | 24.0 | 22.0 | 21.5 | 22.3 | 20.7 | 19.7 | 20.4 | 18.8 | 17.9 | 17.7 | 17.5 |
| **Gynaecological (%)** | 1.5 | 1.7 | 2.1 | 2.1 | 1.9 | 2.0 | 1.9 | 1.7 | 1.6 | 1.6 | 1.3 |
| **Haematological (%)** | 0.5 | 0.4 | 0.3 | 0.3 | 0.3 | 0.3 | 0.4 | 0.3 | 0.2 | 0.3 | 0.2 |
| **Metabolic (%)** | 8.8 | 8.3 | 8.1 | 7.5 | 7.8 | 7.5 | 7.8 | 7.9 | 7.5 | 8.0 | 8.3 |
| **Musculoskeletal/Skin (%)** | 2.4 | 2.3 | 2.3 | 2.3 | 3.2 | 3.7 | 3.7 | 3.5 | 2.9 | 3.3 | 3.3 |
| **Neurological (%)** | 11.8 | 11.8 | 11.7 | 12.3 | 12.5 | 12.9 | 12.7 | 12.9 | 12.8 | 13.0 | 12.4 |
| **Renal/Genitourinary (%)** | 5.4 | 5.2 | 5.3 | 5.4 | 4.7 | 4.6 | 4.6 | 5.1 | 6.5 | 6.0 | 5.5 |
| **Respiratory (%)** | 15.8 | 17.8 | 15.9 | 14.4 | 13.2 | 13.4 | 12.7 | 11.8 | 11.1 | 11.8 | 13.0 |
| **Sepsis* (%)** | 9.0 | 9.8 | 11.4 | 11.7 | 14.1 | 13.6 | 14.1 | 16.8 | 19.1 | 17.8 | 16.9 |
| **Trauma (%)** | 4.2 | 4.2 | 5.1 | 5.5 | 5.3 | 5.0 | 5.1 | 5.2 | 5.3 | 5.5 | 5.6 |
| **Others (%)** | 0.4 | 0.2 | 0.1 | 0.1 | 0.3 | 0.2 | 0.5 | 0.4 | 0.3 | 0.4 | 0.4 |

**Supplementary Table 4 Trends in Admission Diagnosis Category**

Admission diagnosis were categorized into different systems. *Sepsis was coded when patient had proven or suspected infection with ≥2 systemic inflammatory response syndrome criteria.
