# Supplementary figures and images for: Characteristics and outcomes of patients admitted to adult intensive care units in Hong Kong: a population retrospective cohort study from 2008 to 2018
Source: J Intensive Care. 2021 Jan 6;9:2. doi: 10.1186/s40560-020-00513-9 (PMC7788755; doi:10.1186/s40560-020-00513-9)

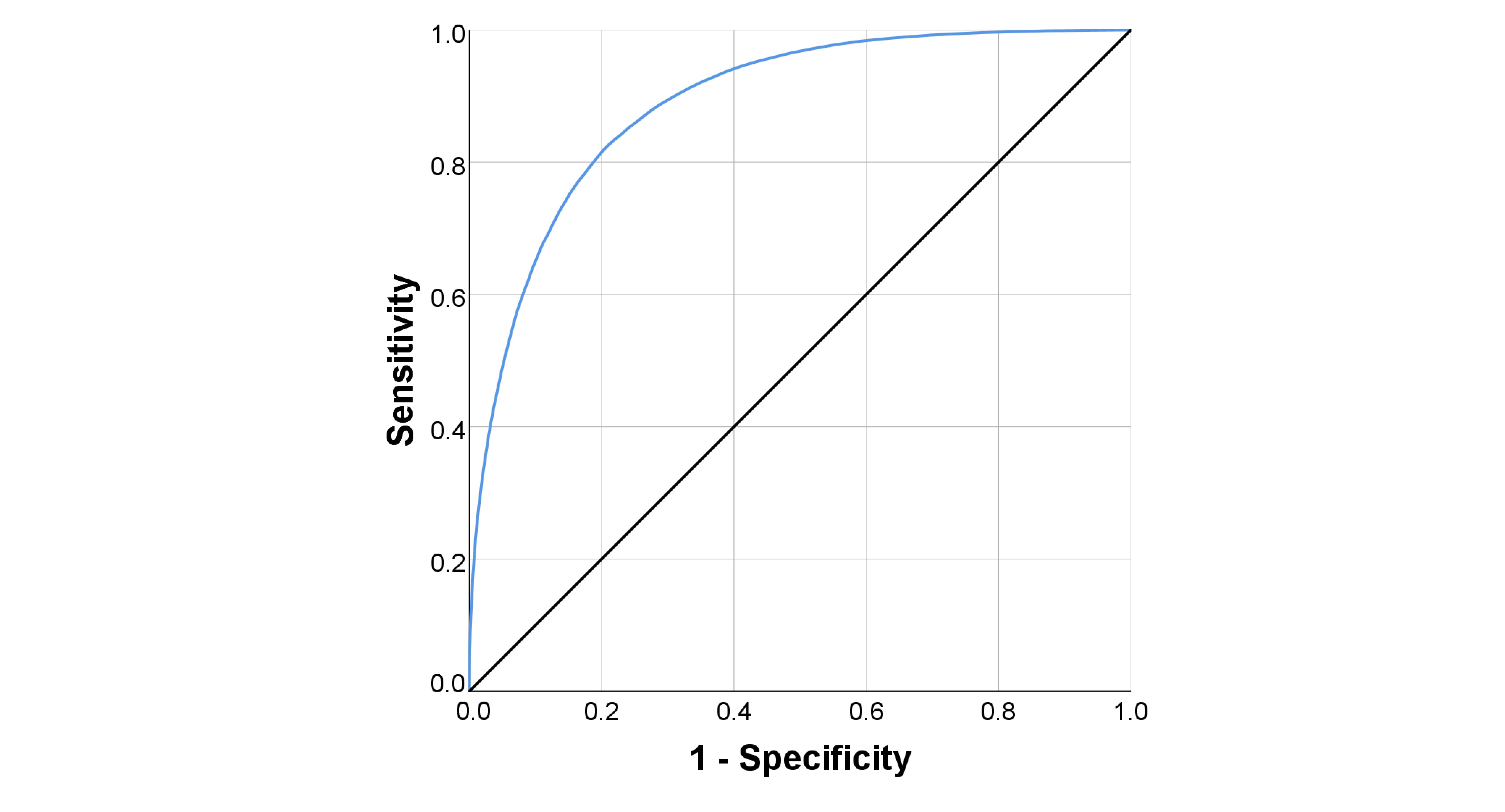

Supplement: Supplementary file 5 — Additional file 5: Supplementary Figure 1. Receiver operating characteristic curve of APACHE IV on Hospital Mortality. Discrimination performance of APACHE IV score on hospital mortality of critically ill patients in Hong Kong. [file 40560_2020_513_MOESM5_ESM.tif]
