# Supplementary material for: Characteristics and outcomes of patients admitted to adult intensive care units in Hong Kong: a population retrospective cohort study from 2008 to 2018
Source: J Intensive Care. 2021 Jan 6;9:2. doi: 10.1186/s40560-020-00513-9 (PMC7788755; doi:10.1186/s40560-020-00513-9)
Supplement: Supplementary file 6 — Additional file 6: Supplementary Table 5. Comparison of APACHE IV Predicted and Observed Hospital Mortality. The study cohort was divided into 10 deciles of APACHE IV predicted mortality groups. Observed number of hospital deaths was compared with APACHE IV predicted number of deaths in each group. 510 patients did not have APACHE IV predicted mortality due to missing data and were not included in this table. APACHE, Acute Physiology and Chronic Health Evaluation. [file 40560_2020_513_MOESM6_ESM.docx]

**Supplementary Table 5 Comparison of APACHE IV Predicted and Observed Hospital Mortality**

| **APACHE IV Predicted Mortality Risk Decile** | **Observed Number of Hospital Deaths** | **Observed Hospital Mortality Rate** | **APACHE IV Predicted Number of Hospital Deaths** | **APACHE IV Predicted Hospital Mortality Rate** | **Total Number of Patients in Risk Decile** | |
| --- | --- | --- | --- | --- | --- | --- |
| 0 – 0.10 | 1090 | 1.7% (95%CI 1.6 – 1.8%) | 2550 | 4.0 (95%CI 4.0 – 4.0%) | 63708 |  |
| 0.11 – 0.20 | 1883 | 9.1 (95%CI 8.7 – 9.5%) | 3113 | 15.0 (95%CI 14.9 – 15.0%) | 20777 |  |
| 0.21 – 0.30 | 2052 | 16.8% (95%CI 16.1 – 17.5%) | 3077 | 25.2 (95%CI 25.1 – 25.2%) | 12225 |  |
| 0.31 – 0.40 | 2120 | 25.7% (95%CI 24.8 – 26.7%) | 2904 | 35.2% (95%CI 35.2 – 35.3%) | 8239 |  |
| 0.41 – 0.50 | 2104 | 33.8% (95%CI 32.6 – 35.0%) | 2821 | 45.3% (95%CI 45.2 – 45.3%) | 6231 |  |
| 0.51 – 0.60 | 2137 | 42.4% (95%CI 41.0 – 43.8%) | 2792 | 55.4% (95%CI 55.3 – 55.4%) | 5043 |  |
| 0.61 – 0.70 | 2154 | 49.2% (95%CI 47.7 – 50.7%) | 2867 | 65.4% (95%CI 65.3 – 65.5%) | 4382 |  |
| 0.71 – 0.80 | 2309 | 58.3% (95%CI 56.7 – 59.8%) | 2989 | 75.4% (95%CI 75.3 – 75.5%) | 3962 |  |
| 0.81 – 0.90 | 2807 | 68.7% (95%CI 67.3 – 70.2%) | 3494 | 85.6% (95%CI 85.5 – 85.7%) | 4083 |  |
| 0.91 – 1.00 | 4071 | 86.7% (95%CI 85.7 – 87.6%) | 4475 | 95.3% (95%CI 95.2 – 95.3%) | 4698 |  |

The study cohort was divided into 10 deciles of APACHE IV predicted mortality groups. Observed number of hospital deaths was compared with APACHE IV predicted number of hospital deaths in each group. 510 patients did not have APACHE IV predicted mortality due to missing data and were not included in this table. APACHE, Acute Physiology and Chronic Health Evaluation.
